# Redox biomarker levels in patients with myelodysplastic syndrome

**DOI:** 10.3892/br.2025.1923

**Published:** 2025-01-14

**Authors:** Eleni Tsiara, Sotiria Makri, Zoi Skaperda, Nikolaos Giannakoulas, George Vasilopoulos, Demetrios Kouretas

**Affiliations:** 1 Department of Hematology, Faculty of Medicine, School of Health Sciences, University of Thessaly, Viopolis, Mezourlo, Larissa 41110, Greece; 2 Department of Biochemistry and Biotechnology, University of Thessaly, Viopolis, Mezourlo, Larissa 41500, Greece

**Keywords:** myelodysplastic syndrome, oxidative stress, biomarker, reduced glutathione, antioxidant activity, lipid peroxidation

## Abstract

Myelodysplastic syndrome (MDS) is a heterogeneous clonal disorder characterized by insufficient hematopoiesis, peripheral blood cytopenia and an increased risk for malignant transformation to acute myeloid leukemia. Several factors, such as age, sex and lifestyle, promote the development of MDS syndrome. Oxidative stress, along with its detrimental effects, cause hematological disorders; however, its role in the pathogenesis of MDS is unknown. The present study enrolled 50 patients with MDS and 50 additional healthy individuals to assess the endogenous antioxidant defense system by measuring specific redox biomarkers at the time of diagnosis. Glutathione (GSH) levels, catalase (CAT) activity and total antioxidant capacity (TAC) were measured in red blood cells, whereas levels of thiobarbituric acid reactive substances (TBARS) and protein carbonyls were measured in the plasma. A decrease in GSH levels, increased TBARS levels and TAC levels were observed in patients with MDS compared with healthy volunteers, supporting the hypothesis that oxidative stress disturbance could promote MDS.

## Introduction

Myelodysplastic syndrome (MDS) refers to various bone marrow ailments that include aberrant blood cell development and function (1). MDS arises from genetic mutations in stem cells that produce blood cells in the bone marrow (2). These mutations decrease proliferation of stem cells, resulting in an insufficient number of normal blood cells. As a result, people with MDS, which primarily affects older adults (up to 70 years), typically have decreased blood flow, which leads to fatigue, weakness, shortness of breath and increased susceptibility to infections (3-5). MDS has been also referred to as pre-leukemia, due to its involvement in progression of acute myeloid leukemia (AML) (2,6).

MDS is caused by dysregulated apoptosis in the hematopoietic compartment, leading to cell death and cytopenia (7). MDS has also been identified as a heterogeneous set of cell maturation malignancies and deregulated innate and inflammatory immune response (8) caused by several genomic events (9). Typically, cytopenia on standard peripheral blood analysis indicates a predisposition to MDS. Anaemia (low haemoglobin and haematocrit values), thrombocytopenia and leukopenia, which are easily diagnosed by a standard complete blood analysis, are indications of myelodysplastic syndromes (10). To ascertain the cellularity and aspirate morphology of bone marrow cells, biopsy is required, and identification of bone marrow blasts (immature blood cells) is key for risk assessment (2). Numerous internationally recognized scoring systems are used to categorize MDS. The most widely used systems are the original International Prognostic Scoring System (IPSS) (11) and Revised IPSS (IPSS-R) (12). Broadly, people with MDS are divided into lower- and higher-risk MDS, according to karyotype, number of blasts and neutrophils and the number of blood platelets (13).

Depending on number of abnormal cells in bone marrow, the proportion of blasts in both blood and bone marrow and the presence of cytogenetic abnormality, the World Health Organization (WHO) classifies MDS into different categories: Refractory anemia (RA) is the most common form of MDS. The resulting low red blood cell count in RA leads to anemia, characterized by weakness, fatigue and shortness of breath. Refractory cytopenia with multilineage dysplasia (RCMD) is another prevalent subtype affecting multiple blood cell lineages, including red and white blood cells and platelets; this subtype accounts for 30% of cases of MDS (5,14). Further subtypes of MDS are RA with excess blasts (RAEB), which has a greater quantity of blasts in the bone marrow and can progress to AML, and MDS with isolated del (5q) (14).

MDS is more prevalent at older age but can sometimes occur in children and young adults (14,15). MDS accounting for less than 5% of hematopoietic neoplasia in childhood (15-17). Age-associated increases in oxidative stress, which interfere with normal tissue function, are caused by a breakdown of antioxidant defense systems and reactive oxygen species (ROS) generation (18,19). Numerous hematological neoplasms, including MDS, are known to be affected by oxidative stress, and there is growing evidence for this in the literature (20-22). The role of ROS in carcinogenesis may be concentration- and time-dependent (23); disruption in ROS levels can cause cell death or accelerate the aging process and age-associated disorders (24). Decreased ROS levels are more likely to accelerate the growth of a tumor via enhanced cancer cell proliferation (23). Hence, controlling ROS levels is key since they serve a critical role in the evolution of tumors and the emergence of tumor-associated pathologies.

The role of oxidative stress in the pathogenesis and development of MDS and AML is achieved via mechanisms such as the accumulation of aberrant and immature blood cells in bone marrow, leading to a greater generation of ROS (25). In a study with 97 patients with MDS, a high level of ROS production in bone marrow cells of patients with MDS and AML was observed (26), which is attributed to iron overload due to required red blood cell transfusion (27). Furthermore, multiple effects on blood cells, including DNA damage, apoptosis (programmed cell death) and impaired differentiation have been observed in patients with MDS (21,28). These effects lead to further accumulation of abnormal blood cells, worsening of anemia and progression to AML (25). For example, when compared with normal cells, the red blood cells and platelets of people with low-risk MDS show lower reduced glutathione (GSH) and elevated ROS levels, respectively (25).

Identification of oxidative stress-related biomarkers is key. The present study aimed to assess the endogenous antioxidant defense system in patients with MDS at diagnosis, without having received any drug therapy or following a specific diet, by measuring redox biomarkers [GSH, catalase (CAT) activity, total antioxidant capacity (TAC) and levels of lipid peroxidation through thiobarbituric acid reactive substances (TBARS) and protein (carbonyl) oxidation. Identifying the redox status of these patients may facilitate future study into the treatment of various disorders, such as MDS.

## Materials and methods

### Study design

A total of 50 adult patients (28 male and 22 females, median age 75 years, age range 45-89 years) diagnosed with MDS from January 2020 to January 2022 were enrolled with a median follow-up of 29 months (range 20-44 months). Patients were classified as those with refractory anemia (RA, 16 males and 16 females with a median age of 75 years), patients with refractory anemia with excess blasts (RAEB, 4 males and 2 female with a median age of 75 years) and patients with refractory cytopenia with multilineage dysplasia (RCMD, 8 males and 6 females with a median age of 75 years). Diagnosis and stratification of MDS was based on WHO criteria (29). An additional 50 age- and sex-matched healthy volunteers (27 males and 23 female; median age 71 years, range 63-80 years) served as an internal control. The samples from two groups were collected at the same time period. Participants were hospitalized or visited the outpatient clinic of the Hematology Department of the General University Hospital of Larissa (Larissa, Greece).

All experimental procedures were approved by the Bioethics Committee of the University of Thessaly (approval no. 47/02.12.2019) and were carried out in conformity with the Helsinki Declaration. The inclusion criteria for the MDS group were as follows: i) Diagnosis of MDS syndrome and ii) consent to participate in the study. The inclusion criteria for healthy individuals were as follows: i) not suffering from any chronic disease, ii) not taking any medication and iii) belonging to the same age group as patients with MDS. If MDS patients have any other form of malignancy except MDS, and if the healthy volunteers had any disease (chronic or autoimmune), they were not included in the study. All participants were mentally competent and provided written informed consent prior to sample acquisition.

### Sample acquisition

A total of 4 ml peripheral blood was drawn from each participant, collected in EDTA tubes, maintained on ice and processed within 4 h. All samples were collected at the time of diagnosis before any treatment. Participants were asked to abstain from alcohol and tobacco use for ≥5 days prior to the sample acquisition.

### Blood separation

Samples were centrifuged for 10 min at 1,380 x g at 4˚C. The plasma supernatant was separated into aliquots for measuring TAC, TBARS and protein carbonyl levels. The rest of the packed erythrocytes were lysed in distilled water (1:1 v/v), agitated and centrifuged at 4,000 x g for 15 min at 4˚C to produce red blood cell lysate foranalysis of GSH levels and CAT activity.

Using a commercial kit (Hemoglobin Drabkin, Dutch Diagnostics; cat. No. 553-820), hemoglobin concentration was determined. A total of 5 µl erythrocyte lysate was combined with 1 ml working hemoglobin reagent (reagent R1, pH 7.3). An incubation for 10 min at RT in the absence of light was performed, and the optical density (OD) of samples was measured at 540 nm. In each experiment, 1 ml R1 was used as blank.

### Redox biomarker assessment. Biomarkers related to antioxidant capacity

*GSH.* GSH concentration was assessed as previously described (30). A total of 400 µl red blood cell lysate was added to 400 µl 5% trichloroacetic acid (TCA), vigorously agitated and centrifuged at 14,500 x g for 5 min at 5˚C. The supernatant was collected and 90 µl 5% TCA was added again to each tube, vigorously agitated, centrifuged at same conditions and clear supernatant was collected. A total of 20 µl supernatant was mixed with 660 µl sodium-potassium phosphate buffer (67 mM, pH 8.0) and 330 µl 5,5'-dithiobis-2 nitrobenzoate (1 mM), samples were incubated for 15 min in the absence of light at room temperature and OD was estimated at 412 nm.

*CAT activity.* Activity of CAT was measured as previously described (19,21). A total of 4 µl red blood cell lysate (diluted in distilled water, 1:10) was added to 2,991 ml sodium-potassium phosphate buffer (67 mM, pH 7.4). Following 15 min incubation at 38˚C, 5 µl 30% H_2_O_2_ was added. The conversion of H_2_O_2_ into H_2_O and O_2_, which causes an alteration in absorbance, was read at 240 nm for 125 sec. CAT activity was calculated based on the molar extinction coefficient of H_2_O_2_ (43.6/M/cm) (31).

*TAC.* TAC was determined as previously described (32). A total of 20 plasma, 480 phosphate buffer (10 mM; pH 7.4) and 500 µl 2,2-diphenyl-1-picrylhydrazyl radical (0.1 mM) solution was incubated in the absence of light at room temperature for 60 min. Samples were centrifuged at 15,000 x g for 5 min at RT and the OD was estimated at 517 nm.

*Biomarkers related to oxidative damage*

*TBARS.* TBARS levels were assessed, as previously described (33). A total of 100 µl plasma and 1 ml mixture of 35% TCA and Tris-HCl (pH 7.41) (1:1) was incubated for 10 min at room temperature. Then, 1 ml Na_2_SO_4_ (2 M) and TBA (55 mM) was added at 95˚C for 45 min. An ice bath was used to cool samples for 4 min at 4˚C, 1 ml 70% TCA was added . Then, 1.5 ml tubes were filled with 1 ml sample and a centrifugation at 11,200 x g for 3 min in RT performed. The OD was identified at 530 nm. TBARS concentration was calculated using the molar extinction coefficient of malondialdehyde (MDA; 155x10^3^/M/cm) (31).

*Protein carbonyls.* Protein carbonylation was assessed as previously described (31). A total of 100 µl 20% TCA and plasma (1:1) was vortexed vigorously and incubated at 4˚C for 15 min, followed by centrifugation at 15,000 x g at 5˚C for 5 min. Supernatant was discarded and the sediment was resuspended in 500 µl 2,4-dinitrophenylhydrazine (DNPH; 10 mM), diluted in 2.5 M HCl. Samples suspended in 500 µl 2.5 M HCl were used as a blank. Following 1 h incubation at room temperature with vortexing every 15 min for 5 sec in the absence of light. Supernatant was thrown away, and a resuspension of the sediments with 1 ml 10% TCA was performed. Samples were centrifuged again under the aforementioned conditions (15,000 x g at 5˚C for 5 min) and sediment was washed three times with 1 ml ethanol-ethyl acetate mixture (1:1 v/v). After resuspending the pellets, samples were centrifuged (15,000 x g at 5˚C for 5 min). Pellets were reconstituted in 1 ml urea (5 M; pH 2.3), after the final wash with ethanol-ethyl acetate mixture (1:1 v/v), vortexed for 5 sec and incubated at 37˚C for 15 min. Following centrifuging (15,000 x g, 5˚C, 3 min), OD of the mixtures was calculated at 375 nm. The molar extinction coefficient of DNPH (22x10^3^/M/cm) served to calculate the protein carbonyl concentration (31).

### Chemicals and equipment

Commercial kits for Hb determination, TCA, H_2_O_2_, solution, DPPH and DNPH were provided by Sigma-Aldrich (Merck KGaA) and DTNB, TBA, Na_2_SO_4_, urea, ethanol and ethyl acetate were provided by Thermo Fisher Scientific, Inc. All spectrophotometrical assays were measured by U-1500 Ultra Violet-Visible spectrophotometer (Hitachi Corporation).

### Statistical analysis

All statistical analyses were conducted with GraphPad Prism, version 8.0.1 (GraphPad Software, Inc.; Dotmatics). Normality of distribution was assessed with Shapiro-Wilk test. For parametric measures, the unpaired t test was used to compare two groups and one-way ANOVA, with Dunn's post hoc test was used for multiple comparisons between control group and different MDS subtypes. Data are presented as the mean ± SEM. P<0.05 was considered to indicate a statistically significant difference. Each assay was performed in triplicates.

## Results

### Lower hemoglobin levels and platelet count in patients with MDS patients

Patients with MDS had statistically significant lower hemoglobin levels and platelet count compared with controls. There was no significant variation in white blood cell count (Table I).

### Classification of MDS subtype

Patients with MDS were classified into the appropriate categories, according to WHO (Table II): 32 patients had RA), 6 out of 50 patients have refractory anemia with excess blasts (RAEB) and 12 have refractory cytopenia with multilineage dysplasia (RCMD).

### Decreased GSH and increased TAC in patients with MDS

**Figure 1:**
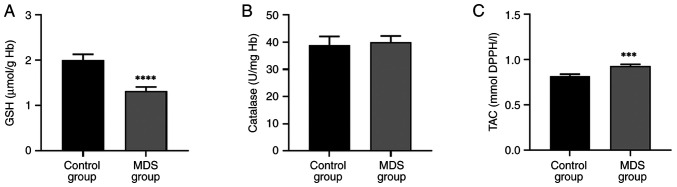
Impact of MDS on biomarkers associated with antioxidant capacity. (A) GSH concentration. (B) Catalase activity. (C) TAC. ^****^P<0.0001, ^***^P<0.001 vs. control. MDS, myelodysplastic syndrome; GSH, reduced glutathione; TAC, total antioxidant capacity; Hb, hemoglobin.

A significant decrease in GSH levels was observed in patients with MDS while TAC significantly increased compared with the control group (Fig. 1A and C). Catalase activity remained unaffected in patients with MDS compared with controls (Fig. 1B).

**Figure 2:**
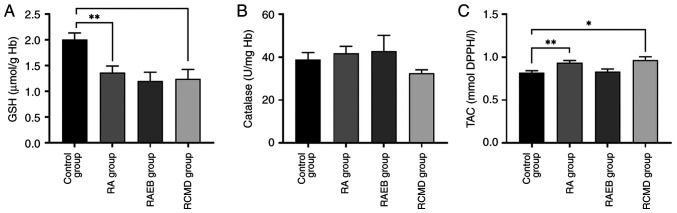
Impact of myelodysplastic syndrome subtype on biomarkers relevant to antioxidant capacity. (A) GSH concentration. (B) Catalase activity. (C) TAC. ^*^P<0.05, ^**^P<0.01. GSH, reduced glutathione; TAC, total antioxidant capacity; Hb, hemoglobin; RA, refractory anemia; RAEB, refractory anemia with excess blasts; RCMD, refractory cytopenia with multilineage dysplasia.

A significant decrease in GSH levels was observed between control and RA and RCMD group (Fig. 2A). TAC significantly increased in RA and RCMD compared with control group (Fig. 2C), while CAT activity remained unaffected (Fig. 2B).

### Increased TBARS levels in patients with MDS

**Figure 3:**
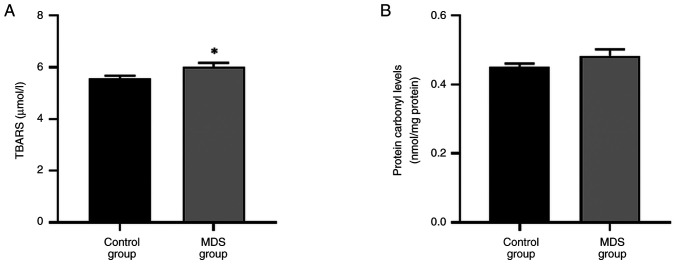
Impact of MDS on biomarkers associated with oxidative damage. (A) TBARS and (B) protein carbonyl levels. ^*^P<0.05 vs. control. MDS, myelodysplastic Syndrome; TBARS, thiobarbituric acid reactive substanses.

A statistically significant increase in the levels of lipid peroxidation, indicated by TBARS, was observed in patients with MDS compared with controls (Fig. 3A), while no statistically significant change in protein carbonyl levels was observed (Fig. 3B).

**Figure 4:**
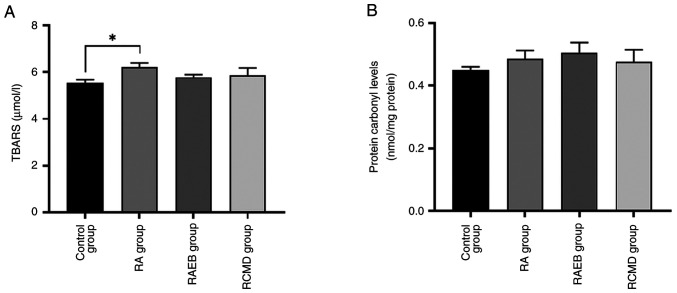
Impact of Myelodysplastic Syndrome subtype on biomarkers relevant to oxidative damage. (A) TBARS and (B) protein carbonyl levels. ^*^P<0.05. RA, refractory anemia; RAEB, refractory anemia with excess blasts; RCMD, refractory cytopenia with multilineage dysplasia; TBARS, thiobarbituric acid reactive substanses.

A significant increase in lipid peroxidation was observed between control and RA group (Fig. 4A), while no changes in protein carbonyl levels were observed (Fig. 4B).

## Discussion

Here, the redox profile of patients with MDS was evaluated by measuring GSH levels, the decomposition rate of H_2_O_2_ through CAT enzyme activity, TAC, lipid peroxidation (TBARS) and protein oxidation levels (protein carbonyls). CAT activity and protein oxidation levels were not significantly affected in patients with MDS compared with the control group. TBARS and TAC levels revealed statistically significant increases, with a concomitant decrease in GSH levels in patients with MDS compared with the control group. CAT activity and protein oxidation levels did not exhibit significant differences between MDS subtypes. TBARS increased significantly in RA group, TAC increased both in RA and RCMD group compared with control, while GSH concentration was significantly higher in RA and RCMD group compared with controls. While the present study indicates that the most affected MDS subtype is RA, the number of patients belonging to the other subtypes (RAEB and RCMD) was inadequate to draw safe conclusions. As a result, further studies with larger sample sizes are needed.

MDS is a heterogeneous group of acquired clonal disorders of hematopoietic progenitor cells, characterized by inefficient hematopoiesis in bone marrow, dysplasia in one or more myeloid lineages, cytopenia in the peripheral blood and an increased risk of progression to AML (34-36). In MDS, bone marrow precursor cells suffer damage and normal hematopoiesis is blocked. The marrow cannot produce enough cells, resulting in cytopenia. Bone marrow is the semi-solid tissue inside the bones. In adults, it is the main hematopoietic organ, producing ~500 billion blood cells/day, which enter the circulation through the vascular network of the marrow (37). The core chambers of long bones and bones in the axial skeleton contain bone marrow, which consists of hematopoietic tissue and adipose cells surrounded by vascular sinuses within a lattice of cancellous bone, representing ~5% of body weight in humans. It is the primary lymphoid tissue and hematopoietic organ, producing erythrocytes, granulocytes, monocytes, lymphocytes and platelets (38).

According to growing evidence of the literature, oxidative stress is associated with development and progression of a wide range of hematological neoplasms (1,20,21). Development of many hematological disorders, including MDS and leukemia, is impacted by chronic oxidative stress (28). Leukemic cells (blasts) survive under oxidative stress and acquire a plethora of mechanisms to shield themselves from stress, including increase of gene expression encoding for antioxidant enzymes (20,39). ROS are critical signaling molecules that are closely involved in the pathophysiology of many types of disease and have an impact on carcinogenesis, including in MDS, AML and chronic myeloid leukemia (28). ROS have been observed to restrict the self-renewal of hematopoietic stem cells, associated with MDS and inefficient hematopoiesis (28,40).

Patients with MDS have high levels of ROS production, leading to oxidative stress, and vice versa (28). Oxidative stress is responsible for damage to key biomolecules, such as DNA, lipid and protein, and also contributes to mitochondrial dysfunction (41). Studies have indicated that patients with MDS present evidence of mitochondrial damage, such as transcriptional, morphological and functional abnormalities (42,43). MDS is also associated with mutations in both nuclear-encoded mitochondrial genes and mitochondrial DNA (42,44). Anemia and ineffective erythropoiesis (the main symptoms of MDS), have also been hypothesized to be associated with MDS due in part to mitochondrial dysfunction (44). The present study did not perform any mitochondrial assessment to determine changes in their function in patients with MDS.

ROS may be involved not only in oxidative damage of DNA, protein and lipids, leading to cell damage (45), but also in pathogenesis and resilience to drugs (20). Although several references report oxidative stress and elevated ROS levels, they only concentrate on biomarkers related to cellular metabolism and do not assess the endogenous antioxidant defense system in patients with MDS (21,28,35). GSH, TBARS and TAC may impact on the prognosis of MDS, since these biomarkers are crucial for the redox status estimation (26,46,47).

GSH is the most abundant endogenous, non-enzymatic antioxidant in the body, so it is a key biomarker for the estimation of the andioxidant potential in both blood and tissues (48). One important factor in MDS is the association between oxidative damage and GSH levels. Patients with MDS have lower intracellular GSH content and higher ROS levels in bone marrow cells, indicating oxidative stress (28). The present findings revealed that GSH levels were significantly decreased in patients with MDS compared with the control group. Additionally, GSH levels were significantly decreased in patients with RA and RMCD. Mechanisms linking MDS with oxidative stress conditions and potentially reduced glutathione levels are mitochondrial DNA mutation, systemic inflammation, bone marrow stromal abnormalities, and mitochondrial dysfunction (35). The majority of the present patients were aged 55-86 years, contributing to the hypothesis that oxidative stress leads to age-associated impairment of normal body function (24). Key parameters associated with alterations in GSH levels are its recycling rate and activity of enzymes involved. Glutathione peroxidase (GPx) and glutathione reductase (GR), are enzymes that convert GSH to the oxidized form (GSSG) and vice versa (49). Therefore, the decrease in GSH in MDS is likely associated with increased activity of GPx, which contributes to its oxidation, converting it to GSSG, and decreased activity of GR, which regenerates it. Moreover, in a 2007 study of 14 patients with MDS, the decrease in GSH levels may also have been due to elevated ROS levels (25): Decreased GSH levels in erythrocytes and platelets and increased ROS levels were detected (25). According to Saigo *et al* (50), such an increase in ROS production is due to overaccumulation of iron molecules in patients with MDS. Conversely, in a recent study by Montes *et al* (20), it was reported that patients with MDS have higher levels of GSH compared with healthy individuals. In the aforementioned study, there was no difference in GSH levels in patients with early and advanced MDS. The findings of the present study indicate the inability to maintain adequate levels of GSH, a key endogenous antioxidant, in MDS. Decreased levels of GSH may be due to either a decrease in activity of enzymes that reduce it or higher amounts of endogenous ROS production.

The course of disease and length of time that patients with MDS survive are influenced by GSH levels. Low GSH levels cause increased oxidative stress in MDS due to increased ROS generation. This may worsen survival rate, cause more cellular damage and accelerate the disease progression (51-53). Furthermore, oxidative stress is linked to cellular apoptosis and DNA damage. This contributes to the inefficient hematopoiesis that characterizes MDS, where bone marrow is unable to produce adequate levels of healthy red blood cells (26). This inefficiency can lead to more severe symptoms and complications, decreasing survival time (51).

As MDS progresses, bone marrow function gradually declines and there is an increased probability of transformation to AML. Inadequate GSH levels intensify oxidative stress, which can accelerate bone marrow dysfunction and raise risk of leukemic transition (54).

The decomposition rate of hydrogen peroxide is determined by activity of enzymes such as peroxidase and CAT (55). CAT converts H_2_O_2_, which is a potentially reactive form of oxygen, into H_2_O and O_2_. In the present study, no significant change in catalase enzyme activity was observed between healthy individuals and patients with MDS. A statistical significant difference was also not observed between the MDS subtypes and the control. However, in a recent study, an increase in CAT activity was observed in MDS-affected people overall and when these patients were divided according to the stage of disease (20).

TAC levels were statistically significantly higher in patients with MDS, compared with control group. TAC was elevated in RA and RMCD compared with controls. In a study in 2019, in patients with a variety of neoplastic diseases, including MDS, a negative correlation was observed between TAC and lipid peroxidation levels (22).

TAC represents the capacity of blood plasma to eliminate free radicals. Every blood component has an antioxidant effect that contributes differently to TAC, which functions as a general measure of overall antioxidant status (56). Therefore, TAC may be influenced by various factors, making it less specific as a biomarker than GSH or TBARS. Further investigation is needed to validate TAC function as a prognostic marker in MDS. Proteins and amino acids, under oxidative stress are damaged, irreversibly generating carbonylated proteins. Here, no significant variation was observed in protein oxidation levels in MDS compared with healthy participants. In a study conducted in Spain involving patients with MDS aged 70 years, no significant change in protein carbonyl levels was observed (20). Conversely, in the aforementioned study, when participants were separated into early and advanced MDS, patients with early MDS had significantly reduced protein oxidation levels, suggesting protection from oxidative stress impacts (20). On the other hand, in a study with 32 patients with MDS, carbonylated protein levels were considerably higher in RARS and RMCD group compared with controls (45).

Lipid peroxidation was statistically significantly higher in patients with MDS compared with healthy controls. Polyunsaturated fatty acids are converted into active and unstable lipid peroxides under oxidative stress. MDA is the product of lipid oxidation, therefore TBARS are reported as equivalents of MDA, accordingly (49). Serological and molecular markers in patients with MDS are associated with an increased concentration of MDA and therefore elevated levels of TBARS and lipid peroxidation (35). In patients with MDS, a significant increase in TBARS levels is observed with a concomitant elevation in ROS levels and a significant decrease in TAC (22). A recent study observed a significant decrease in TBARS levels in patients with early and advanced MDS (20). However, in the aforementioned study, a concurrent increase in GSH endogenous levels and rate of hydrogen peroxide degradation via the activity of CAT was observed in patients with MDS, suggesting that these patients developed antioxidant defense mechanisms that could shield them from oxidative damage associated with MDS (20,35).

Anemia and iron overload, which affect the majority of patients with MDS, are hypothesized to be negative independent prognostic factors linked to a higher risk of leukemic transformation and shorter survival time (46,57). As aforementioned, lipid peroxidation, which can inhibit hematopoietic stem cell self-renewal and directly cause DNA damage and genomic instability, is brought on by an excess of ROS (38,46). Elevated levels of TBARS, as observed in the present study, indicate oxidative stress, which can damage critical biomolecules (49).

It is widely acknowledged that redox status biomarkers are prognostically important in various types of neoplastic disease, including MDS (22,51). Older patients with MDS have higher levels of oxidative stress than healthy people (22). The findings of the present study are consistent with those of earlier studies in which biomarkers (TBARS and protein carbonyls) associated with oxidative damage were increased, while antioxidant capacity-associated markers (GSH, CAT and TAC) were either decreased or unchanged (22,54). Oxidative stress may be involved in the development of MDS. Patients with MDS do not have properly developed antioxidant mechanisms to cope with such pathological conditions (25,50). Antioxidants could be included everyday diet as a potential remedy; polyphenol-rich plant extracts prevent DNA damage caused by peroxyl radicals and enhance the overall redox profile (58). For example, tannic acid, a common tannin found in red wines, tea and coffee, administerred in male C3H mice with liver neoplasms results in a decrease in the overall incidence of hepatic tumors (59).

All biomarkers evaluated in the present study are a first step to a comprehensive strategy for assessing redox status of individuals (47). All present patients with MDS had a mild hematological profile during the time of their disease prognosis (i.e. low hemoglobin and hematocrit values). As maintained by WHO, patients diagnosed with myelodysplastic syndrome who have moderate anaemia are classified as low-risk and they have prolonged survival and delayed disease progression. Median follow-up length was 29 months, which is not long enough to definitively address whether patients with low-risk myelodisplastic abnormality have prolonged survival and delayed disease progression. Consequently, the present study did not determine the association between redox biomarkers (GSH, TAC, TBARS) and prognosis in patients with MDS.

The present study revealed a disturbance of oxidative redox homeostasis in patients with MDS compared with healthy controls, which was substantiated by a decrease in GSH and increase in TBARS levels. To the best of our knowledge, the present study is the first to assess redox biomarkers in MDS subtypes and controls. Subtype analysis revealed that the most affected MDS type was RA, but more studies with a larger number of patients are needed to validate this. These observations indicate a possible manifestation of oxidative damage in the blood of patients with MDS via the disruption of the antioxidant defense system based on key antioxidant molecules. The development of lipid peroxidation and the drop in GSH levels demonstrated this. The increase in TAC may serve as an adaptive mechanism of antioxidant defense, but without protecting the patients against MDS, which was expressed through the promotion of plasma lipid peroxidation levels in MDS group. Future research is required to define the role that redox biomarkers serve in the pathogenesis and clinical course of MDS and whether adding antioxidants, such as vitamin E, ascorbic acid or iron chelators, to the regular diets of patients with MDS improves their redox profile. These substances may combat myelodisplastic disorder via promotion of hematopoiesis that is normally hindered along with the progression of MDS.

## Figures and Tables

**Table I:** Blood parameters.

Parameter	Control group	MDS group
Hemoglobin, g/l	13.70±0.16	11.50±0.23^a^
Platelet count, x10^9^/l	240.80±8.34	188.30±17.30^b^
White blood cell count, x10^9^/l	5.50±0.19	9.07±2.00

^a^P<0.0001,

^b^P<0.05 vs. control. MDS, myelodysplastic syndrome.

**Table II:** Classification of patients with MDS.

MDS subtype	Number of patients
Refractory anemia	32
Refractory anemia with excess blasts	56
Refractory cytopenia with multilineage dysplasia	12

MDS, myelodysplastic syndrome.

## Data Availability

The data generated in the present study may be requested from the corresponding author.
